# Endoreduplication as a part of flower ontogeny in *Trifolium pratense* cultivars

**DOI:** 10.1186/s40529-016-0150-x

**Published:** 2016-10-27

**Authors:** Valéria Kocová, Nikola Straková, Vladislav Kolarčik, Albert Rákai, Pavol Mártonfi

**Affiliations:** 1 Department of Botany, Faculty of Science, Institute of Biology and Ecology, P.J. Šafárik University, Mánesova 23, 04154 Košice, Slovakia; 2 Krosnianska 69, 04022 Košice, Slovakia

**Keywords:** Endoreduplication, *Trifolium pratense*, Flower ontogeny, Ploidy level

## Abstract

**Background:**

Endoreduplication appears in numerous plant species and plays a vital role during ontogeny. The presence of polyploid cells in an otherwise diploid organism is tied specifically to the taxonomy, ecology and physiology of the studied specimen. Little is known about the changes in endopolyploidy levels of floral organs during their development. In order to uncover the workings of endoreduplication in polysomatic species, our study examines flowers of *T. pratense* in three ontogenetic stages by means of flow cytometry.

**Results:**

Cultivar ‘Manuela’ is characterized by the presence of 2C–8C and ‘Dajana’ 2C–16C nuclei. In general, the frequencies of nuclei only slightly changed during development. Endopolyploidy levels represented by endoreduplication index (EI) in the ‘Manuela’ sepals and stamens showed statistical differences between young and old stages, other organs of both cultivars between stages are not statistically different. Significant differences between ‘Manuela’ and ‘Dajana’ cultivars were found only in sepals of I. stage, and in petals and carpels of III. stage. Cultivars showed a similar pattern of endopolyploidy. However, a considerable decrease in EI ‘Manuela’ petals and carpels at III. stage was detected as opposed to ‘Dajana’. Overall, a higher endoreduplication index is distinctive for organs of the ‘Dajana’ cultivar.

**Conclusions:**

In this study we prove the permanent presence of endopolyploid cells in the floral organs of *T. pratense* throughout their development.

## Background

The phenomenon of endopolyploidy is very common in eukaryotic organisms and is distributed across the whole plant kingdom (Barow 2006). It results from endoreduplication. Under certain conditions this process causes a cell to switch from the mitotic to the endoreduplication cycle (Carvalheira 2000; Joubés and Chevalier 2000; Barow and Meister 2003; Leitch et al. 2013). Endoreduplication is characterized by the lack of M-phase of the cell cycle leading to several repeated rounds of DNA synthesis. Thus, the DNA content of a cell multiplies (Barow 2006; Barow and Jovtchev 2007).

The true biological significance of this phenomenon is not yet fully understood (Kudo and Kimura 2002b; Kudo et al. 2004; Leitch et al. 2013; Kron 2015) and new challenges still appear (Trávníček et al. 2015). There are some indications that endoreduplication is involved in the early process of growth and differentiation (Kondorosi et al. 2000). It is often present in cells with high metabolic activity and progressive growth (Cebolla et al. 1999), therefore it implies its role in the enlargement of the size of the cell and serves as a trigger for organ growth (Bourdon et al. 2012). Agricultural crops such as *Lycopersicon esculentum* (Smulders et al. 1994), *Raphanus sativus* (Kudo and Kimura 2002a), *Solanum tuberosum* (Ujtewaal 1987), *Zea mays* (Engelen-Eigles et al. 2000) and ornamental species (e.g. *Dianthus caryophyllus*, Agulló-Antón et al. 2013) are endopolyploid. This suggests potential of endopolyploidy alterations in the improvement of growth in these plants.

The occurrence of endopolyploidy has commonly been studied in vegetative organs (Barow and Meister 2003; Yang and Loh 2004; Sliwinska and Lukaszewska 2005; Kolano et al. 2008; Bainard et al. 2012; Straková et al. 2014), but quite rarely in floral organs of seed plants (Barow and Meister 2003), except for a few detailed studies of orchid flowers (Kudo and Kimura 2001a; Lee et al. 2004; Jean et al. 2011; Trávníček et al. 2015), carnations (Agulló-Antón et al. 2013) and clovers (Kocová et al. 2014). All these studies showed that endopolyploidy is organ- and tissue- specific. Generally, cotyledons, petioles and lower leaves are characterized by higher degrees of endopolyploidy, while roots, upper leaves and floral organs usually show much lower degrees (Barow 2006).

The level of endopolyploidy varies during the individual organ development, e.g. in seedlings (*Brassica oleracea*, Kudo and Kimura 2001b; *Allium fistulosum*, Kudo et al. 2003; *Beta vulgaris*, Sliwinska and Lukaszewska 2005; *Chenopodium quinoa*, Kolano et al. 2008; and *Trifolium pratense*, Straková et al. 2014), young leaves and root parts (*Spathoglottis plicata*, Yang and Loh 2004), or storage organs and nutrient tissues (*Lycopersicon esculentum*, Bergervoet et al. 1996; *Capsicum annum*, Ogawa et al. 2012 and *Sorghum bicolor*, Kladnik et al. 2006), which implies a role of endoreduplication in the developmental process.

Endopolyploidy is related to its taxonomic position in phylogenetic systems (Barow and Meister 2003). The Fabaceae is considered as a family with a very high frequency of endopolyploid species (Barow and Meister 2003; Barow and Jovtchev 2007) including the genus *Trifolium* (Barow and Meister 2003; Kocová and Mártonfi 2011; Kocová et al. 2014) whose phylogeny and species relationship was thoroughly studied (Ellison et al. 2006). Previous research on the species *Trifolium pratense* (Straková et al. 2014) showed how the endopolyploidy level varies during the development of organs in seedlings. Endopolyploidy in floral organs during ontogeny is researched here to complete the overall picture of endopolyploidy in *T. pratense*. This report significantly contributes to the research on endopolyploidy, since there is not much knowledge about developmental changes in endopolyploidy levels of floral organs and only a limited number of research studies on petal development have been published so far, e.g. on cabbage (Kudo and Kimura 2002b), carnations (Agulló-Antón et al. 2013) and orchids (Lee et al. 2004).

## Methods

### Plant material

**Fig. 1:**
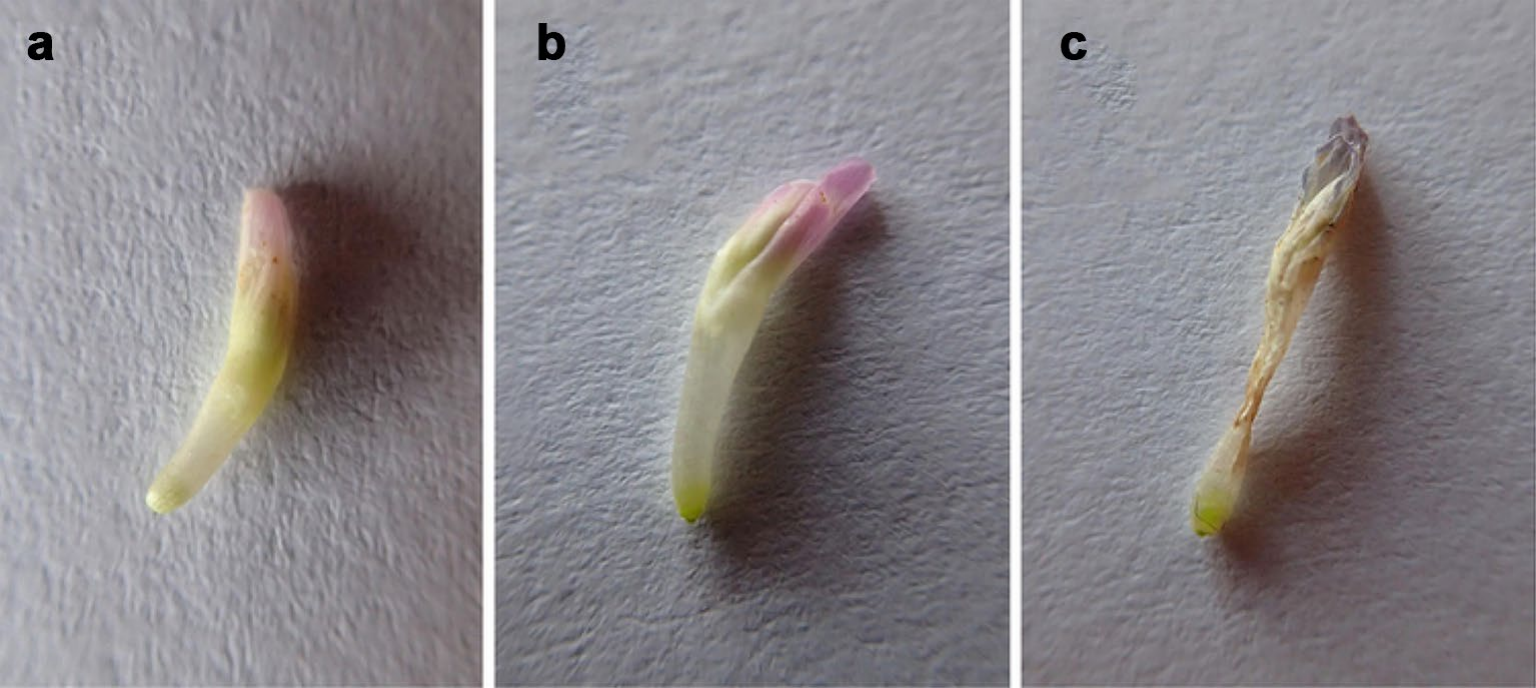
Developmental stages of floral organs of *Trifolium pratense* L.: stage I-**a**, stage II-**b**, stage III-**c**

Seeds of *Trifolium pratense* L., commercial cultivars ‘Manuela’ and ‘Dajana’ cultivated in laboratories were germinated in sand for approximately 3 weeks under controlled conditions, 12 h photoperiod (12 h day, 12 h darkness), 28/20 °C temperature and 60% humidity. After germination, seedlings were transferred into pots with a slightly modified Hoagland solution (Hoagland and Amon 1950) which contained macronutrients [(g/l) 0.9517 Ca(NO_3_)_2_, 0.06 NH_4_H_2_PO_4_, 0.6106 KNO_3_, 0.4905 MgSO_4_·7H_2_O], 1 mL solution of micronutrients [(g/l) 0.5983 H_3_BO_3_, 0.4016 MnCl_2_·4H_2_O, 0.0903 ZnSO_4_·7H_2_O, 0.0524 CuSO_4_·5H_2_O, 0.0204 CoCl_2_·6H_2_O, 0.0288 Na_2_MoO_4_] and 1 mL solution of Fe [(g/l) 26.1 EDTA, 16.1568 KOH, 24.9 FeSO_4_.7H_2_O]. Plants were cultivated under controlled conditions with a 16/8 photoperiod (16 h day, 8 h darkness), 28/20 °C temperature and 60% humidity. Endopolyploidy was estimated in the following organs: sepals, petals, stamens and carpels. The flower ontogeny was divided into three stages (I–III, Fig. 1). The first stage was characterized by completely closed flowers (Fig. 1a), second stage flowers were fully opened (Fig. 1b) and at stage III the flowers bore/exhibited signs of aging (wilting, Fig. 1c).

### Karyological analysis

For chromosome number counting, seeds of *Trifolium pratense* ‘Manuela’ were germinated in a Petri dish on a wet filter paper at room temperature. After two days, roots approximately 2–5 mm long were collected. The root tips were pre-treated in 0.002 M aqueous solution of 8-hydroxyquinoline for 4 h, fixed in acetic ethanol (acetic acid and 97% ethanol in the ratio 1:3), washed with distilled water and hydrolysed for 5 min in 1 N HCl at 60 °C. The root tip meristems were washed in distilled water again and then squashed in 45% acetic acid using the cellophane square technique (Murín 1960). After that the slides were stained with 10% aqueous solution of Giemsa stock solution and after 24 h they were washed in distilled water and air-dried. Chromosomes were counted in a drop of immersion oil at 100× using a Leica DM 2500 microscope equipped with a DFC 290 HD camera, using the Leica ver. 3 application suite software.

### Flow cytometry preparation

We used flow cytometry (FCM) for high speed genome analysis, measuring the fluorescence of stained nuclei in our samples. We followed the standard 3 day method (Greilhuber and Obermayer 1997) using the leaves of *Trifolium pratense* ‘Manuela’ and ‘Dajana’. As an internal reference standard *Solanum lycopersicum* ‘Stupicke polní tyčkové raní’ (2C DNA content = 1.96 pg, Doležel et al. 1992) was used. A small piece of a fresh leaf of *T. pratense* and of the reference standard were chopped together with a sharp razor blade in a Petri dish containing 1 ml ice-cold GPB (general purpose buffer: 0.5 mM spermine × 4 HCl, 30 mM sodium citrate, 20 mM MOPS [MOPS = 4-morpholine propane sulfonate], 80 mM KCl, 20 mM NaCl, 0.5% [v/v] Triton X-100, pH 7.0, according to Loureiro et al. (2007). The suspension was filtered through a 42 μm nylon mesh and each sample was stained separately using 10 μg propidium iodide (PI), 10 μg RNase and 2 μl β-mercaptoethanol. For the DNA content estimation of the isolated nuclei the Partec CyFlow ML flow cytometer with green solid state laser (532 nm wavelength and 150 mW) and band-pass 590 nm optical filter was used. The data were plotted on a linear scale and processed with the FloMax 2.70 software. At least 1300 nuclei were measured for the standard and sample peaks and only measurements with coefficients of variation below 5% were used. To evaluate the histograms, the WinMDI 2.9 application was used (Trotter 2000).

Samples for the endopolyploidy analysis were prepared from the floral organs of two *Trifolium pratense* cultivars in three ontogenetic stages (I–III). 15 flowers were removed from each inflorescence. Pooled organs of the same type were co-chopped (15 per sample) following the same procedure as in the genome size measurements. In this case, no reference standard was added. The data for the determination of endopolyploidy were displayed on a logarithmic scale. At least 5000 nuclei were counted for each sample. Processing of the data and the histograms were evaluated as stated above.

### Calculation of genome size

Data visualized by histograms using the flow cytometry software were used for genome size estimations. The calculation of the genome size was made as follows (Doležel and Bartoš 2005):

2C DNA content of the sample = mean peak position of the 2C sample/mean peak position of the 2C reference standard × 2C DNA content of the reference standard (pg).

### Calculation of endopolyploidy

The degree of endopolyploidization is quantified by a parameter called the endoreduplication index (EI) (Bainard et al. 2012), previously known as a cycle value (Barow and Meister 2003) which indicates the mean number of endoreduplication cycles per nucleus. Endoreduplication index can be calculated as follows (Bainard et al. 2012; Barow and Meister 2003):

$${\text{EI }} = {{\left( {0 \, \times {\text{ n}}_{{2{\text{C}}}} + { 1 } \times {\text{ n}}_{{ 4 {\text{C}}}} + { 2 } \times {\text{ n}}_{{ 8 {\text{C}}}} + { 3 } \times {\text{ n}}_{{ 1 6 {\text{C}}}} \ldots } \right)} \mathord{\left/ {\vphantom {{\left( {0 \, \times {\text{ n}}_{{2{\text{C}}}} + { 1 } \times {\text{ n}}_{{ 4 {\text{C}}}} + { 2 } \times {\text{ n}}_{{ 8 {\text{C}}}} + { 3 } \times {\text{ n}}_{{ 1 6 {\text{C}}}} \ldots } \right)} {\left( {{\text{n}}_{{ 2 {\text{C}}}} + {\text{ n}}_{{ 4 {\text{C}}}} + {\text{ n}}_{{ 8 {\text{C}}}} + {\text{ n}}_{{ 1 6 {\text{C}}}} \ldots } \right)}}} \right. \kern-0pt} {\left( {{\text{n}}_{{ 2 {\text{C}}}} + {\text{ n}}_{{ 4 {\text{C}}}} + {\text{ n}}_{{ 8 {\text{C}}}} + {\text{ n}}_{{ 1 6 {\text{C}}}} \ldots } \right)}}$$

where n_2C_, n_4C_, n_8C_, n_16C_, … indicate the counts of nuclei of corresponding C-levels (2C, 4C, 8C, 16C…). Samples with the EI higher than 0.1 are considered endopolyploid (Barow and Meister 2003).

### Statistical methods

Calculated endopolyploidy index data were subjected to statistical analyses of mean or median similarity. Assumptions of parametric tests, data normality and homogeneity of variance were tested using *Shapiro*–*Wilk* and *Levene* test (or *F* test for equal variance when two groups were compared), respectively. The statistical analysis *ANOVA* (one-way analysis of variance) was used to determine the differences among corresponding organs and individual developmental stages. If the assumptions of *ANOVA* were not met a non-parametric, alternative *Kruskal*–*Wallis* test was employed. Pairwise comparisons were then performed with *Tukey’s* or *Mann*–*Whitney* (with *Bonferroni corrected p values* to determine significance) pairwise post hoc tests. When comparing two groups (comparisons between cultivars), a t test was used instead, in case of unequal variances p value was determined using a permutation test. All tests were carried out as implemented in Past 3.10 software (Hammer et al. 2001).

## Results

**Fig. 2:**
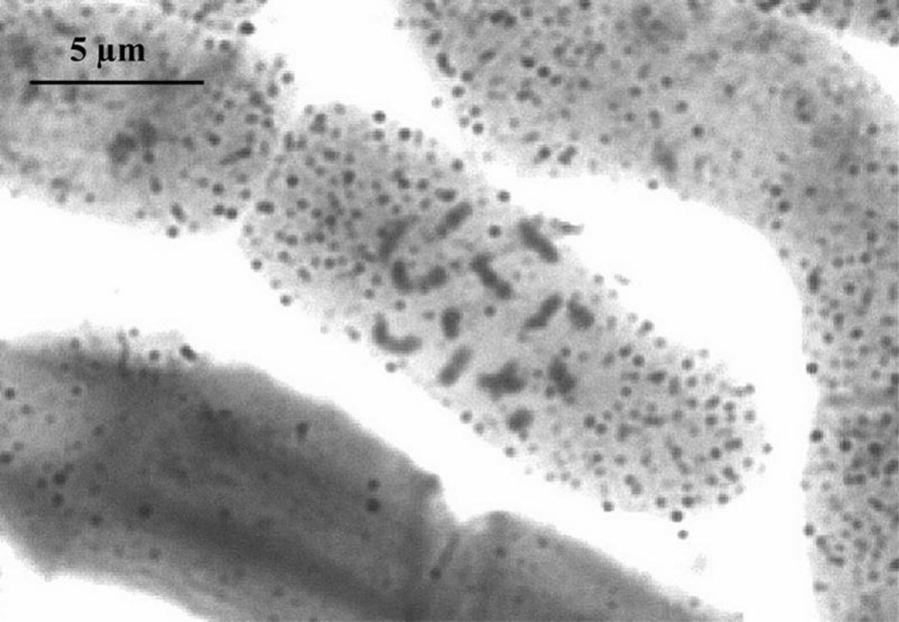
Somatic chromosomes of *Trifolium pratense* L. (2n = 2x = 14)

The investigated plants of *T. pratense* were confirmed to be diploids with 2n = 2x = 14 chromosomes (Fig. 2). The genome size is approximately 2C = 0.93 pg in both ‘Manuela’ and ‘Dajana’ cultivars.

**Fig. 3:**
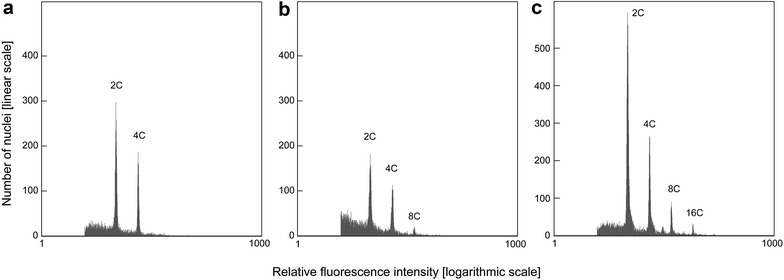
Histogram examples with typical FCM records showing different ploidy levels of ‘Dajana’plants. **a** Petals in II stage. **b** Carpels in II stage. **c** Carpels in III stage

**Fig. 4:**
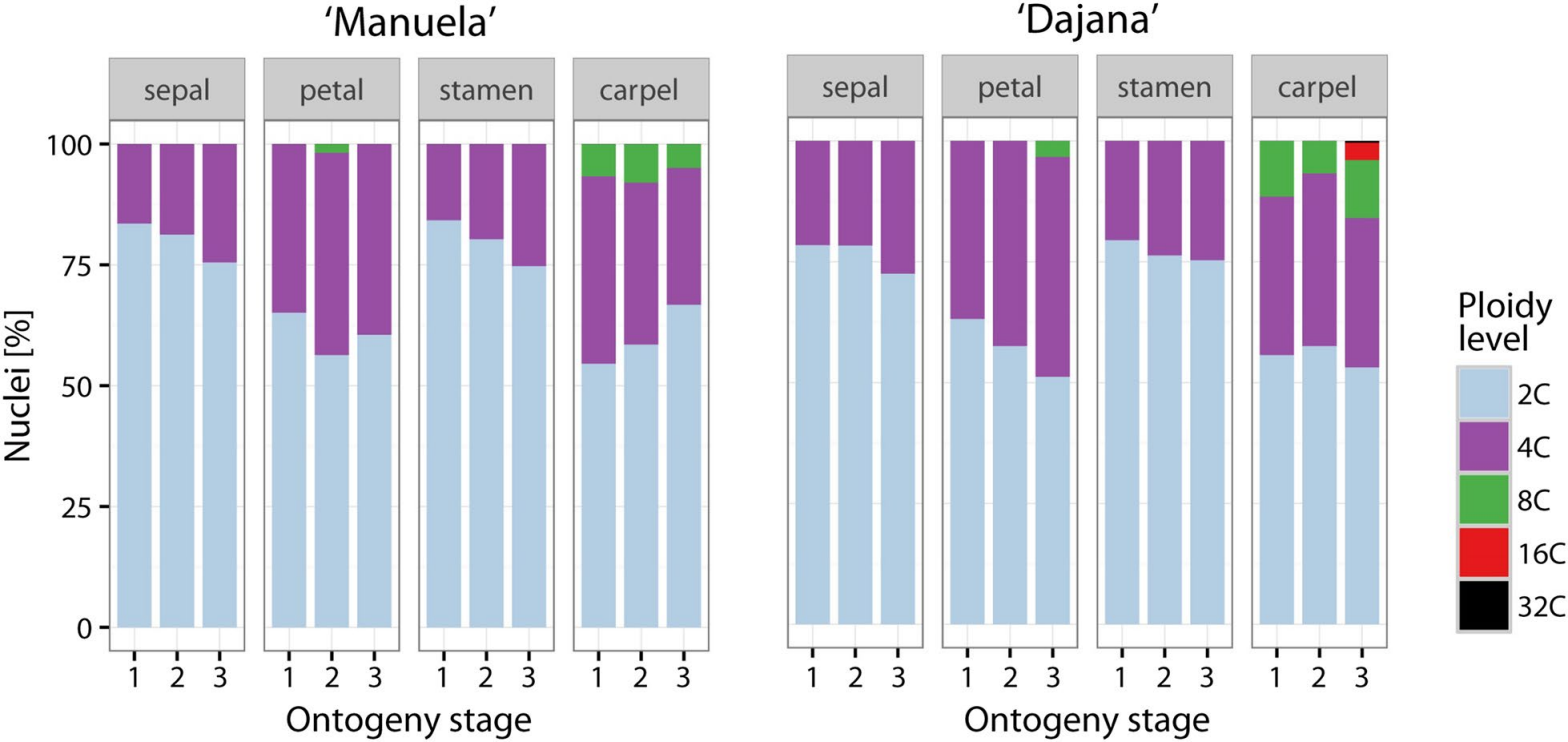
Polysomaty of *T. pratense* flowers. Frequencies of floral organs nuclei of ‘Manuela’ and ‘Dajana’ in three ontogenetic stages

Mixoploidy was found in each measured sample of all investigated floral organs (Fig. 3), ranging from 2C + 4C to 2C − 8C nuclei in ‘Manuela’ and from 2C + 4C to 2C − 16C nuclei in ‘Dajana’ (only a single case showed up to 2C–32C; Fig. 4). The proportion of nuclei of particular ploidy levels differs among floral organs in both cultivars. In the sepals and stamens only 2C and 4C nuclei were present. The latter within range of mean values 15.7–27.4% across both organs, three stages and both cultivars. In contrast, higher frequencies of 4C nuclei (28.3–45.5%) were found in both petals and carpels and 8C nuclei in petals (less than 4% in the II. and III. stage of ‘Manuela’ and ‘Dajana’ respectively) and carpels (4.9–11.9% in all stages). In conclusion, only negligible changes in the nuclei frequency occur during the three ontogeny stages in the studied organs. More notably, in ‘Dajana’ carpels of the III. stage 16C and 32C nuclei were present, but the latter were found only in a single sample and determined by a low number of nuclei. Only minimal differences in the nuclei proportion were observed between both investigated cultivars (Fig. 4). A substantial difference was detected in petals, ‘Manuela’ had 8C nuclei already in the II. stage, whereas ‘Dajana’ contained these nuclei only in the III. ontogenetic stage. An apparent difference among cultivars is the maximum ploidy level achieved; while it was 8C in ‘Manuela’, there were additional 16C and even 32C nuclei recorded in ‘Dajana’.

The endopolyploidy level expressed by EI values, varies significantly among the floral organs (ANOVA, p < 0.05, Table 1). Generally, higher EI of petals and carpels in comparison with EI of sepals and stamens were found (Table 1). Furthermore, significantly different EI values were observed between carpels and petals in the I. stage of both cultivars. Overall, the highest EI value was recorded in the carpels (0.52 in the I. stage in ‘Manuela’; 0.67 in the III. stage in ‘Dajana’) and the lowest one in the stamens (0.16 in the I. stage in ‘Manuela’; 0.21 in the I. stage in ‘Dajana’).

**Table 1:** Summary data of endopolyploidy in *Trifolium pratense* cultivars ‘Manuela’ and ‘Dajana’ in three different ontogeny stages

			Stage I	Stage II		Stage III	
Cultivars	Type of organ	N	EI	N	EI	N	EI
‘Manuela’	*Sepal*	12	0.16 ± 0.04a	9	0.19 ± 0.08a	7	0.24 ± 0.07a
	*Petal*	12	0.35 ± 0.06b	8	0.45 ± 0.13bc	7	0.39 ± 0.06b
	*Stamen*	6	0.16 ± 0.03a	4	0.2 ± 0.04ab	4	0.25 ± 0.05ab
	*Carpel*	11	0.52 ± 0.12c	9	0.49 ± 0.06c	6	0.38 ± 0.11b
‘Dajana’	*Sepal*	6	0.22 ± 0.05a	18	0.22 ± 0.05a	6	0.27 ± 0.04a
	*Petal*	6	0.37 ± 0.05b	17	0.42 ± 0.07b	6	0.52 ± 0.13b
	*Stamen*	4	0.21 ± 0.05a	15	0.24 ± 0.08a	5	0.25 ± 0.07a
	*Carpel*	6	0.56 ± 0.04c	18	0.49 ± 0.11b	6	0.67 ± 0.17b

Values of EI are mean ± standard deviation. Different letters within a column indicate significant differences between organs within one stage at *p* < *0.05;* both cultivars were treated separately in the statistics

*N* number of samples, *EI* endoreduplication index

**Fig. 5:**
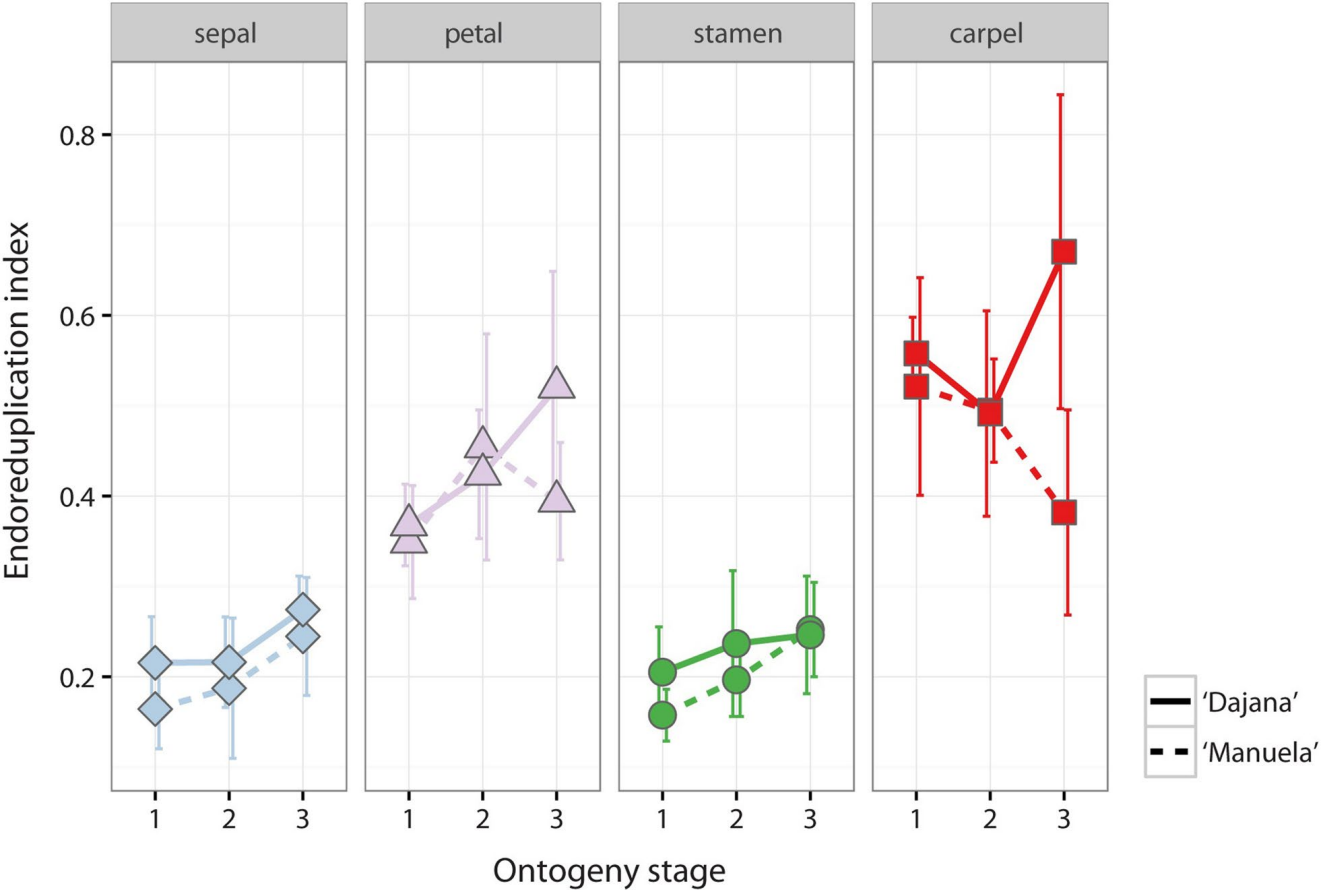
Endoreduplication indexes of the individual floral organs of *Trifolium pratense* L. ‘Manuela’ and ‘Dajana’ during three ontogenetic stages (*bars* represents standard deviation)

Considering ontogenetic stages within floral organs, the statistical analysis (ANOVA) only showed significant differences in EI (p < 0.05) between stage I and stage III of the ‘Manuela’ sepals and stamens (Fig. 5). Other differences were insignificant mainly due to relatively high variation among measured samples. Generally, the EI grows gradually with the ontogenetic stage (Fig. 5). However, some exceptions were noticed. While the EI of the ‘Dajana’ petals increased continuously, a slight drop of EI in the III. stage of the ‘Manuela’ petals was observed. In the ‘Dajana’ carpels the EI in the I. stage was 0.56, then decreased to 0.49 and increased to 0.67 in the II. and III. stage respectively. The rising pattern is broken only in ‘Manuela’ carpels where the EI decreases from 0.52 in the I. stage to 0.38 in the III. stage. The endopolyploidy pattern is very similar in both cultivars, significant differences were present only in sepals of stage I, and petals of stage III (t test, p < 0.05) and carpels of stage III (t test, p < 0.01).

## Discussion

The number of endocycles in cells is genetically predetermined and the degree of endopolyploidy is subject to the systematic position of the given species (Barow and Meister 2003; Bainard et al. 2012). Only certain families comprise more genera and species with polysomatic organs and tissues (Barow 2006). The presence of endopolyploidy is predominantly found in the family Fabaceae and its genus *Trifolium*.

Endopolyploid cells were detected in almost all of the investigated organs of *T. pratense* (Kocová and Mártonfi 2011). In this paper we confirmed endopolyploidy in floral organs and uncovered slight differences in overall endopolyploidy levels during flower development. The typical C-level profile of individual floral organs of *T. pratense* cultivars was found rather similar to the previously analyzed wild type *T. pratense* (2C–8C) (Kocová and Mártonfi 2011). This could be accounted for by genetic predisposition in species, and the slight difference recorded between each cultivar and the wild type could result from minor genetic differences or the impact of other external factors (e.g. growing conditions). The endopolyploidy in flowers occurs in many plant groups (Kudo and Kimura 2001a; Fukai et al. 2002; Lee et al. 2004), but in some, even endopolyploid species, e.g. *Spathoglottis plicata*, endopolyploidy in flowers was not confirmed (Yang and Loh 2004).

Very little is known about the endopolyploidy level during the development of floral organs and its purpose so far. An increasing proportion of endoreduplicated nuclei was detected in the orchid flower development (Lee et al. 2004) as well as in the development of cabbage (Kudo and Kimura 2002b) and carnation petals (Agulló-Antón et al. 2013). We observed a similar situation, although, it seems that the C-values are notably more conserved in the floral organs of *T. pratense* in comparison with the previous studies. This difference is probably rooted in the species specific features of the genus and a unique developmental pathway of floral organs. We found only subtle (tough still detectable) differences in endopolyploidy levels (EI) of the floral organs between specified stages of the flower development. In general, an increasing tendency in endopolyploidy expression paired with development was detected in the floral organs of *T. pratense* (Fig. 5). Such trend is also related to other types of organs and tissues of plants, e.g. seedlings (Sliwinska and Lukaszewska 2005; Kolano et al. 2008), endosperm (Kladnik et al. 2006), and pericarp (Bergervoet et al. 1996; Ogawa et al. 2012). Endoreduplication is involved in the growth of cells and the development of organs as documented by Kladnik et al. (2006), who stated that cell size and endoreduplication are correlated in *Sorghum* endosperm. Lee et al. (2004) reported that flower fresh weight is positively correlated with average C-value.

It seems that the endopolyploidy level of the floral organs in *T. pratense* (Fig. 4) might stabilize close to maturation as seen in orchids (Lee et al. 2004) but the overall EI change in relation to ontogeny was not wholly distinct in our case. However, in contrast to our study, where a continuing change in the endopolyploidy level was recorded even after the maturation of flowers, Lee et al. (2004) found out that in the fully developed flowers of orchids endopolyploidization stops and its ploidy level stabilizes with no further change in the nuclei proportions. Considering this, a difference in the floral life cycle between the two mentioned cases must be noted. Orchid flowers have increased longevity compared to *Trifolium*, which usually turns to wilting quite rapidly. Changes in EI of petals and carpels could thus be explained by continuous flower development (petals, opening of corolla in the II. stage and senescent wilting in the III. stage; carpels, its likely onset of change to pericarp wall or presence of developed seeds). It is not yet fully understood how endopolyploidy affects developmental processes in sepals, stamens and carpels. Kudo and Kimura (2002b) propose, that the obvious importance of changes in the nuclei size from 2C–8C up to 4C–32C in the proximal epidermal cells of petals in comparison with the distal part of petals (a slight increase of 4C nuclei), inevitably results in bud opening. This can be the main reason for the ploidy level increase in petal cells.

Despite a very similar pattern in the endopolyploid profile of the floral organs across flower development found in both ‘Manuela’ and ‘Dajana’ cultivars, slight differences were detected. Most apparent is the increase in EI in petals and carpels in ‘Dajana’ when flowers shift to the third stage and its decrease in the same stage within ‘Manuela’. This may be the result of developmental process differences between both cultivars, which were not investigated in the present study. Slight differences in endopolyploidy levels in cultivars of the same species are likely, as exemplified by research on carnations (Agulló-Antón et al. 2013).

Although the main aim of this study was to determine the changes in endopolyploidy levels in organs during flower ontogeny, other valuable points can be made based on our data. The floral organs in this study showed endopolyploidy patterns similar to the wild type *T. pratense* and other species of the genus, *T. repens* and *T. montanum* (Kocová et al. 2014) (similar mean EI of organs, except that of stamens of natural *T. pratense*, which was 0.41). In conclusion, common characteristics of the *Trifolium* species (Kocová and Mártonfi 2011; Kocová et al. 2014) and *T. pratense* cultivars are (i) EI of petals is always higher than EI of sepals and (ii) the highest EI is found in carpels in comparison with the remaining floral organs. Hence, these seem to be common features in the genus *Trifolium*. However, this rule should not be applied to species of other families (Barow and Meister 2003; Chen et al. 2011). In addition, endopolyploidy patterns differ in comparison with other *Fabaceae* species belonging to different genera such as *Pisum sativum* and *Vicia faba*, although the higher EI of petals over sepals corresponds to cases in *Trifolium*, carpels of the mentioned species were the ones with the lowest EI (Barow and Meister 2003). Putative inconsistency in endopolyploid patterns of the floral organs of different species does not result from coincidence, but is most probably determined by interplay between intrinsic (genetic, cytological, life form) and extrinsic (environmental) factors. It is well known that the endopolyploidy level of organs/tissues is mostly correlated with the phylogenetic placement of the species which is strongly associated with many phenotypic traits (Bainard et al. 2012).

## Conclusions

Endoreduplication serves as the speeding process of the development and growth of floral organs, even though the changes in endopolyploidy of flowers of *T. pratense* during their development are almost negligible. Thus, we assume, while endopolyploidy definitely plays its role during the developmental process of *T. pratense* flowers, the quantitative changes do not speak of significant differences. Recent studies (Kocová and Mártonfi 2011; Straková et al. 2014; this study) provide a comprehensive insight into the endoreduplication pattern of *T. pratense*, thus proving permanent presence of endoreduplication in the life of plants, starting with seedlings throughout the aging of flowers.
